# Different Responses to Premature Atrial Complexes During Wide QRS Complex Tachycardia: What Is the Mechanism?

**DOI:** 10.19102/icrm.2026.17046

**Published:** 2026-04-15

**Authors:** Ahmet Korkmaz, Ozcan Ozeke, Elif Hande Ozcan Cetin, Meryem Kara, İdriz Merovci, Can Ozkan, Firat Ozcan, Serkan Cay, Dursun Aras, Serkan Topaloglu

**Affiliations:** 1 Department of Cardiology, Health Sciences University, Ankara Bilkent City Hospital, Ankara, Turkey; 2 Department of Cardiology, University Clinical Center of Kosovo, Prishtina, Kosovo; 3 Department of Cardiology, Health Sciences University, Bursa City Hospital, Bursa, Turkey; 4 Department of Cardiology, İstanbul Medipol University, İstanbul, Turkey

**Keywords:** Nodofascicular, nodoventricular, right bundle branch block tachycardias

## Abstract

A wide complex tachycardia may result from supraventricular tachycardia (SVT) with bundle branch block (pre-existing or tachycardia-related), SVT with atrioventricular conduction over an accessory pathway (AP), or ventricular tachycardia (VT). The timing of PACs and their effect on tachycardia can help differentiate between atrioventricular nodal re-entry tachycardia variants and AP-mediated tachycardias. A proximal coronary sinus recording is critical for evaluating septal refractoriness and excluding nodal tachycardia and other forms of nodal bystander pre-excitation (such as nodoventricular or nodofascicular re-entrant tachycardia).

## Case presentation

**Figure 1:**
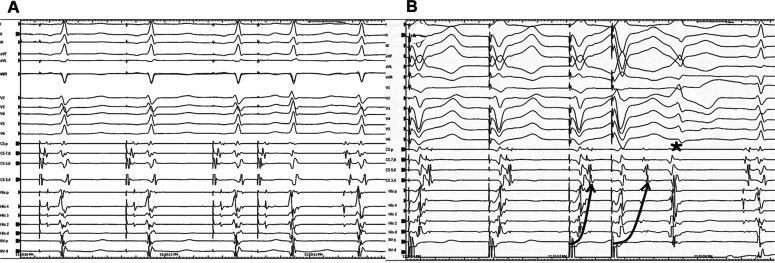
The A–H interval was short and normalized with programmed atrial stimulation **(A)**. Programmed ventricular stimulation **(B)** demonstrates retrograde right bundle branch block conduction and a ventricular echo beat exhibiting a right bundle branch block morphology.

**Figure 2:**
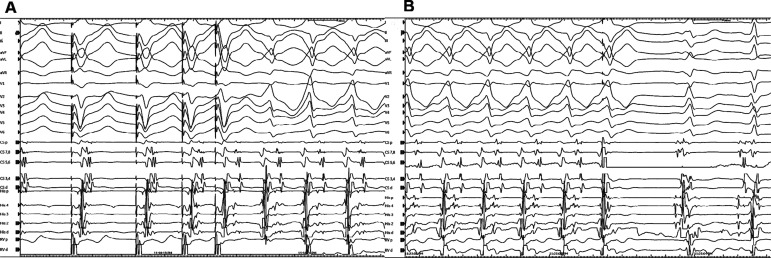
Pre-excited tachycardia was inducible only by ventricular stimulation **(A)** and stoppable by the “early” premature atrial stimulation **(B)**. Note the differences in QRS morphology and H–V intervals following the termination of tachycardia by the “early” premature atrial contraction.

**Figure 3:**
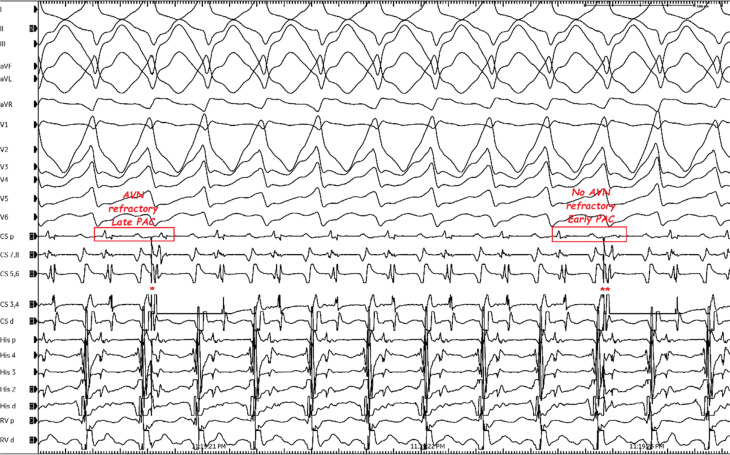
Ventricular activation could not be advanced by “late” premature atrial contractions delivered from the coronary sinus distal electrode (single red star, on the left corner) but was advanced by only “early” premature atrial contractions delivered from the same electrode without changing the QRS morphology (double red star, on the right corner). Note that the morphology of the one antidromic echo beat by programmed ventricular stimulation **(Figure 1B)** was the same as that of tachycardia **(Figure 3)**. A proximal coronary sinus recording is critical for evaluating septal refractoriness and excluding nodal tachycardia and other forms of nodal bystander pre-excitation (such as nodofascicular or nodoventricular re-entrant tachycardia) (compare the first and second rectangular boxes with regard to coronary sinus proximal electrogram morphology and cycle length). *Abbreviations:* AVN, atrioventricular node; PAC, premature atrial contraction.

A 23-year-old woman with a history of catheter ablation of both paraseptal accessory pathway (AP) and typical atrioventricular (AV) nodal re-entrant tachycardia (AVNRT) was referred for a redo ablation. A 12-lead electrocardiogram showed minimal pre-excitation. During the electrophysiology study, programmed atrial and ventricular stimulation responses were obtained **(Figure 1)**. Pre-excited tachycardia was inducible only by ventricular stimulation and terminable by premature atrial contraction (PAC) **(Figure 2)**. Responses to two different PACs from the lateral left atrium are shown in **Figure 3**. What is the mechanism of the tachycardia?

## Discussion

The differential diagnosis of a regular wide QRS tachycardia with 1:1 AV association includes (1) orthodromic re-entrant tachycardia with aberrancy; (2) atrial flutter or atrial tachycardia with ventricular pre-excitation; (3) antidromic re-entrant tachycardia (ART) with retrograde conduction through the bundle branch–His–AV node axis; (4) pre-excited tachycardia due to pathway-to-pathway (duodromic) conduction; (5) AVNRT with bystander AP conduction; (6) orthodromic re-entrant tachycardia with bystander activation of ventricles using another pathway; (7) ventricular tachycardia or bundle branch re-entry tachycardia; (8) junctional tachycardia with aberrancy or fasciculoventricular connection; and (9) antidromic atriofascicular (AF), nodofascicular (NF), or nodoventricular (NV) re-entrant tachycardia (NFRT or NVRT).^1–3^ Several clinical characteristics are useful for the appropriate diagnosis of a wide QRS tachycardia. In 1988, Tchou et al. reported the role of a late atrial premature complex in differentiating AF from NF fibers in a single patient.^4^ The timing of PACs and their effect on tachycardia can help differentiate between AVNRT variants and AP-mediated tachycardias.^5^ A proximal coronary sinus (CS) recording is critical for evaluating septal refractoriness and excluding nodal tachycardia and other forms of nodal bystander pre-excitation (such as NFRT or NVRT).^5,6^ Indeed, both proximal CS activation and His recordings can be used as markers of nodal origin; however, because the His catheter does not consistently provide atrial electrograms, we used the proximal CS catheter as a surrogate marker of nodal activation.

**Figure 4:**
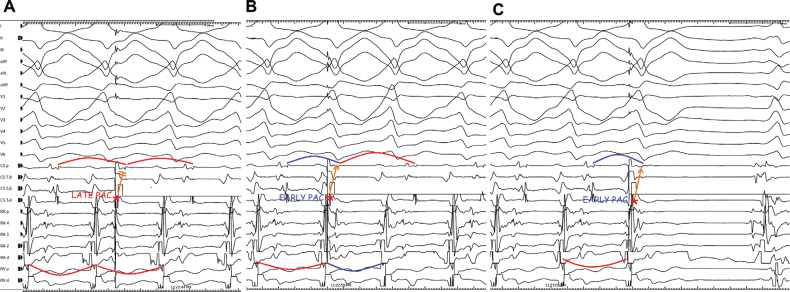
Three different responses to “late” **(A)** and “early” **(B and C)** premature atrial contractions (PACs). Whereas the ventricular activation could not be advanced by “late” PAC delivered from the coronary sinus distal electrode **(A)**, it was advanced by only “early” PAC delivered from the same electrode without changing the morphology **(B)**, and then finally tachycardia showed a termination response to the “early” PAC **(C)**. *Abbreviation:* PAC, premature atrial contraction.

In the current case, a wide complex tachycardia was convincingly demonstrated to be antidromic. Interestingly, it was induced only from the V. The question is whether this WCT was ART or NFRT/NVRT. The essential difference is that the A is required in the former and the latter is independent of the A. However, the programmed atrial stimulation did not show progressive H–V shortening excluding the ART **(Figure 1A)**. Moreover, in the presence of ART, a ventricular advancement response would be expected with both early and late PACs. As only early, but not late, PACs reset the tachycardia, this suggests a re-entry mechanism involving a slow pathway with the diagnosis of antidromic NFRT/NVRT. Distinguishing between antidromic NFRT/NVRT and the bystander participation of an NF fiber during atypical AVNRT is challenging. An AV node-dependent tachycardia (such as AVNRT) with bystander pre-excitation is less likely, as the tachycardia circuit lies close to the His–Purkinje system; thus, one would expect a greater contribution of ventricular depolarization through the conduction system, resulting in a less pre-excited QRS morphology. In the current case, the identical morphology of the ventricular echo beat **(Figure 1B)** and the WCT **(Figure 2B)**, together with the absence of dynamic fusion during the WCT and the resetting response to an early PAC **(Figures 3 and 4)**, does not confirm but supports an active antidromic NFRT/NVRT mechanism. Finally, early PACs advanced and terminated the tachycardia without QRS fusion, confirming the participation of a decremental NF/NV pathway in the antidromic re-entry circuit. Ablation of the slow pathway eliminated inducibility, supporting the nodal origin.
